# Associations between cardiometabolic indices and the risk of diabetic kidney disease in patients with type 2 diabetes

**DOI:** 10.1186/s12933-024-02228-9

**Published:** 2024-04-25

**Authors:** Han Yan, Qing Zhou, Yaqiong Wang, Yifan Tu, Yuxin Zhao, Jie Yu, Kuangyang Chen, Yepeng Hu, Qiao Zhou, Wen Zhang, Chao Zheng

**Affiliations:** 1 Department of Endocrinology, The Second Affiliated Hospital, School of Medicine, Zhejiang University, Hangzhou, 310009 China; 2 Department of Cardiology, State Key Laboratory of Cardiovascular Disease, National Center for Cardiovascular Diseases, Fuwai Hospital, Graduate School of Peking Union Medical College, Chinese Academy of Medical Sciences and Peking Union Medical College, Beijing, 100037 China

**Keywords:** Diabetes kidney disease, Atherogenic index of plasma, Stress hyperglycemia, Insulin resistance

## Abstract

**Background:**

This study was designed to assess the associations between emerging cardiometabolic indices—the atherogenic index of plasma (AIP), the stress hyperglycemia ratio (SHR), the triglyceride-glucose (TyG) index, and the homeostasis model assessment of insulin resistance (HOMA-IR)—and the incidence of diabetic kidney disease (DKD) in type 2 diabetes (T2D) patients.

**Methods:**

We consecutively enrolled 4351 T2D patients. The AIP, SHR, TyG index, and HOMA-IR were calculated from baseline parameters. DKD was defined as a urine albumin/creatinine ratio > 30 mg/g or an eGFR < 60 mL/min per 1.73 m. All participants were categorized into tertiles based on the cardiometabolic indices. Multivariate logistic regression models, restricted cubic splines, and receiver operating characteristic (ROC) curves were used for analysis.

**Results:**

A total of 1371 (31.5%) patients were diagnosed with DKD. A restricted cubic spline showed a J-shaped association of the AIP and TyG index with DKD, a log-shaped association between HOMA-IR and DKD, and a U-shaped association between the SHR and DKD incidence. Multivariate logistic regression revealed that individuals in the highest tertile of the four cardiometabolic indices had a significantly greater risk of DKD than did those in the lowest tertile (AIP: OR = 1.08, 95% CI = 1.02–1.14, *P* = 0.005; SHR: OR = 1.42, 95% CI = 1.12–1.81, *P* = 0.004; TyG index: OR = 1.86, 95% CI = 1.42–2.45, *P* < 0.001; HOMA-IR: OR = 2.24, 95% CI = 1.52–3.30, *P* < 0.001). The receiver operating characteristic curves showed that the HOMA-IR score was better than other indices at predicting the risk of DKD, with an optimal cutoff of 3.532.

**Conclusions:**

Elevated AIP, SHR, TyG index and HOMA-IR are associated with a greater risk of DKD in patients with T2D. Among these indices, the HOMA-IR score demonstrated the strongest association with and predictive value for DKD incidence.

**Supplementary Information:**

The online version contains supplementary material available at 10.1186/s12933-024-02228-9.

## Background

Diabetic kidney disease (DKD) represents a significant and prevalent complication in patients with type 2 diabetes (T2D) [1] and contributes substantially to the global burden of chronic kidney disease (CKD) [2]. The intricate interplay of metabolic disturbances, vascular dysfunction, and prolonged hyperglycemia renders individuals with T2D particularly susceptible to renal complications [3]. Identifying reliable predictors of DKD is imperative for early intervention and targeted therapeutic strategies. In this pursuit, emerging cardiometabolic indices have garnered attention as potential markers reflecting the complex metabolic milieu associated with T2D.

The atherogenic index of plasma (AIP), stress hyperglycemia ratio (SHR), triglyceride-glucose (TyG) index, and homeostasis model assessment of insulin resistance (HOMA-IR) are key indices that encapsulate diverse aspects of metabolic health [4–7]. The AIP, derived from the logarithm of the ratio of triglycerides to high-density lipoprotein cholesterol (HDL-C), offers insights into lipid metabolism and atherogenesis [4]. The SHR, a dynamic metric used to assess the relationship between fasting plasma glucose (FPG) and glycated hemoglobin (HbA1c), reflects the impact of stress-induced hyperglycemia [5]. The TyG index, which is calculated from fasting triglyceride and glucose levels, serves as a surrogate marker for insulin resistance (IR) and metabolic syndrome [6]. HOMA-IR, a well-established measure, quantifies insulin resistance, offering a snapshot of the intricate balance between insulin sensitivity and glucose homeostasis [7]. Although these indices have been shown to correlate with the progression of cardiovascular diseases in T2D patients [8–10], their specific relationships with the development of DKD remain incompletely elucidated. Understanding the contributions of the AIP, SHR, TyG index, and HOMA-IR to the incidence of DKD holds promise for refining risk stratification and guiding targeted therapeutic interventions in individuals with T2DM.

In this context, our study was designed to systematically evaluate the associations between each cardiometabolic index—the AIP, SHR, TyG, and HOMA-IR—and the incidence of DKD in a well-defined cohort of patients with type 2 diabetes. We investigated the intricate relationships between these biomarkers and the progression of diabetic kidney disease. The findings from this investigation may have important implications for risk stratification, early intervention, and personalized management strategies tailored to mitigating the burden of DKD in individuals with type 2 diabetes.

## Methods

### Study design and participants

**Fig. 1:**
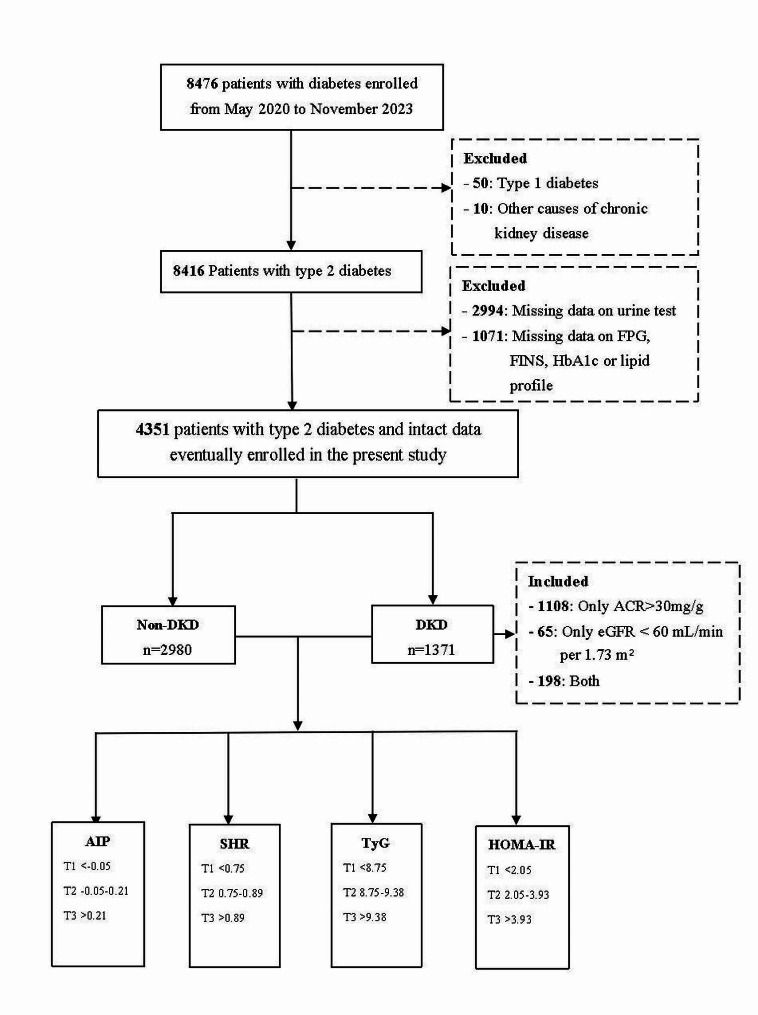
Flowchart of population enrollment FPG, fasting plasma glucose; FINS, fasting insulin; DKD, diabetic kidney disease; AIP, atherogenic index of plasma; SHR, stress hyperglycemia ratio; TyG, triglyceride-glucose; HOMA-IR, homeostasis model assessment of insulin resistance

We prospectively enrolled 8476 consecutive patients admitted to the Second Affiliated Hospital of Zhejiang University for diabetes diagnosis from May 1, 2020, to November 30, 2023. Diabetes status was defined as a FPG ≥ 126 mg/dL, a two-hour oral glucose tolerance test value ≥ 200 mg/dL, an HbA1c ≥ 6.5%, or currently receiving hypoglycemic therapy. Patients with type 1 diabetes, other causes of chronic kidney disease (interstitial nephritis and nephrosclerosis), or missing essential laboratory data were excluded. Comprehensive details of population enrollment are provided in Fig. 1. The study adhered to the Declaration of Helsinki and received authorization from the Second Affiliated Hospital of Zhejiang University Ethics Review Committee (approval number: 2020394). Appropriate consent and assent were obtained from all participants.

### Data collection and endpoint definitions

Baseline demographic and clinical data, including age, sex, vital signs, obesity-related indices, laboratory test results, comorbidities, and medication history, were extracted from an electronic medical recording system by trained physicians. Blood samples were collected between 6:00 am and 10:00 am after an overnight fast of at least 8 h and processed in the laboratory department. The measurements included FPG, fasting insulin (FINS), HbA1c, hemoglobin (HGB), alanine aminotransferase (ALT), aspartate aminotransferase (AST), alkaline phosphatase (ALP), γ-glutamyl transpeptidase (γ-GT), albumin (Alb), blood urea nitrogen (BUN), total triglyceride (TG), total cholesterol (TC), high-density lipoprotein cholesterol (HDL-C), high-sensitivity C-reactive protein (hs-CRP), low-density lipoprotein cholesterol (LDL-C), free triiodothyronine (FT3), and free thyroxine (FT4). Urine samples were collected from patients who were under quiet conditions and free from fever, infection, or other inflammatory conditions. Each patient underwent two urine tests to confirm the presence of albuminuria. The urine albumin/creatinine ratio (ACR) was calculated. The estimated glomerular filtration rate (eGFR) was determined using the Chronic Kidney Disease Epidemiology Collaboration (CKD-EPI) equation for “Asian origin” [11]. DKD was defined as an ACR greater than 30 mg/g or an eGFR < 60 mL/min per 1.73 m², as recommended by the American Diabetes Association [12]. The medication history, encompassing the use of insulin, metformin, sodium-glucose cotransporter-2 inhibitors (SGLT2i), glucagon-like peptide 1 receptor agonists (GLP-1 RA), dipeptidyl peptidase 4 inhibitors (DPP4i), angiotensin-converting enzyme inhibitors (ACEIs), angiotensin receptor blockers (ARBs), beta-blockers, and statins, was also recorded.

### Cardiometabolic biomarker calculations

AIP = log10 [TG (mmol/L)/HDL-C (mmol/L)] [4].

SHR = first FPG (mg/dl)/[(28.7 × HbA1c%) – 46.7] [5].

TyG index = ln [FPG (mg/dl) × TG (mg/dl)/2] [6].

HOMA-IR = [FPG (mmol/L) × FINS (µU/ml)]/22.5 [7].

### Statistical analyses

Continuous variables with a normal distribution are presented as the mean ± standard deviation, and nonnormally distributed data are presented as the median and interquartile range (IQR). Student’s t tests, Wilcoxon rank sum tests, and chi-square tests were utilized to assess parameter differences between DKD patients and non-DKD patients. Participants were categorized into tertiles based on their AIP (T1 < -0.05, T20.05-0.21, T3 > 0.21), SHR (T1 < 0.75, T2 0.75–0.89, T3 > 0.89), TyG index (T1 < 8.75, T2 8.75–9.38, T3 > 9.38), and HOMA-IR (T1 < 2.05, T2 2.05–3.93, T3 > 3.93). Multivariate logistic regression models with three progressive stages of adjustment were used to evaluate associations between cardiometabolic index tertiles and DKD incidence. Model 1 was adjusted for age and sex; Model 2 was adjusted for variables in Model 1 plus body mass index (BMI), systolic blood pressure (SBP), HGB, hyperlipidemia, history of coronary heart disease and stroke; Model 3 was adjusted for variables in Model 2 plus the use of ACEI/ARB, insulin, metformin, SGLT2i, and GLP-1 RA. Trend tests were conducted by including the cardiometabolic indices tertiles in the model as ordinal variables and calculating the Wald statistic. Additionally, restricted cubic spline (RCS) analyses, with five knots placed at the 5th, 27.5th, 50th, 72.5th, and 95th percentiles, were performed to examine the associations between cardiometabolic indices and DKD incidence. Subgroup analyses were also conducted to explore associations between patients with different characteristics, including age, sex, BMI, coronary heart disease, hyperlipidemia, and hypertension. Diagnostic performance was assessed by multivariate ROC analysis. The area under the receiver operating characteristic curve (AUROC) of the cardiometabolic indices was compared using DeLong’s method. The optimal cutoff values of the indices were identified by receiver operating characteristic (ROC) analysis using Youden’s index. Spearman correlation test and partial correlation test (controlling for age, BMI and SBP) were used to analyze the relationship between the indices and hs-CRP. Statistical analyses were carried out using SPSS Statistics (version 26; SPSS, Chicago, IL) and R (version 4.2.0), with a significance threshold set at *P* < 0.05 (two-tailed).

## Results

### Patient characteristics

A total of 4351 patients who were diagnosed with T2D met the inclusion criteria and participated in the study. The mean age of the population was 53.8 ± 14.1 years, and 1510 (34.7%) were female. Of the enrolled patients, 1371 were diagnosed with DKD during hospitalization. Among the 1371 patients with DKD, 1108 were diagnosed solely because of albuminuria, 65 were diagnosed solely because of eGFR < 60, and 198 had both albuminuria and eGFR < 60. Table 1 outlines the baseline characteristics of the patients, categorized by the presence or absence of DKD. Compared to patients without DKD, those with DKD tended to be older, have higher blood pressure, and be more obese. Furthermore, FPG, FINS, and HbA1c levels were elevated in DKD patients. Notably, DKD patients exhibited greater AIP, SHR, TyG index, and HOMA-IR values than non-DKD patients:

AIP—DKD patients, mean 0.19 [IQR − 0.03, 0.45]; non-DKD patients 0.09 [-0.11, 0.31]; SHR—DKD patients 0.82 [0.65, 0.97]; non-DKD patients 0.81 [0.67, 0.93]; TyG index—DKD patients 9.46 [8.91, 10.10]; non-DKD patients 9.12 [8.66, 9.64]; HOMA-IR—DKD patients 3.87 [2.36, 6.44]; non-DKD patients 2.80 [1.70, 4.67])

**Table 1:** Baseline characteristics of patients with and without diabetic kidney disease

Characteristics	Overall	Non-DKD	DKD	P value
	(*n* = 4351)	(*n* = 2980)	(*n* = 1371)	
Age, years	53.8 ± 14.1	53.0 ± 13.9	55.7 ± 14.8	< 0.001
Female	1510 (34.7)	1007 (33.8)	503 (36.7)	0.064
SBP, mmHg	129 ± 17	126 ± 15	135 ± 19	< 0.001
DBP, mmHg	79 ± 10	77 ± 10	81 ± 11	< 0.001
Heart rate, beats/min	82 ± 13	81 ± 12	85 ± 13	< 0.001
Obesity-related index				
Body mass index	24.6 ± 6.0	24.4 ± 6.6	25.1 ± 4.3	< 0.001
Head circumference, cm	55.7 ± 3.4	55.7 ± 3.5	55.7 ± 3.3	0.910
Neck circumference, cm	38.8 ± 3.9	38.6 ± 3.8	39.1 ± 3.9	0.003
Waist circumference, cm	90.7 ± 10.7	90.0 ± 10.4	92.2 ± 11.3	< 0.001
Hip circumference, cm	97.3 ± 8.6	97.1 ± 8.2	97.8 ± 9.3	0.127
Visceral fat, cm^2^	95.9 ± 38.6	93.2 ± 37.5	102 ± 40.3	< 0.001
Subcutaneous fat, cm^2^	181 ± 65.2	179 ± 65.9	186 ± 63.5	0.005
Laboratory test				
FPG, mmol/L	8.1 [6.5, 11.1]	7.8 [6.4, 10.4]	9.0 [6.9, 12.4]	< 0.001
FINS, pmol/L	54.5 [34.4, 87.2]	51.7 [32.7, 80.4]	62.5 [39.8, 102.0]	< 0.001
HbA1C, %	8.86 ± 2.30	8.62 ± 2.25	9.39 ± 2.34	< 0.001
HGB, g/L	140 ± 19.1	143 ± 17.4	136 ± 21.4	< 0.001
ALT, U/L	22.0 [15.0, 34.0]	22.0 [16.0, 34.0]	22.0 [15.0, 34.0]	0.283
AST, U/L	21.0 [17.0, 28.0]	21.0 [17.0, 27.0]	22.0 [18.0, 28.0]	0.433
ALP, U/L	77.0 [64.0, 93.0]	75.0 [62.0, 91.0]	81.0 [67.0, 98.0]	< 0.001
γ-GT, U/L	26.0 [17.0, 42.2]	25.0 [17.0, 40.0]	28.0 [19.0, 49.0]	< 0.001
Alb, g/L	41.8 ± 4.58	42.4 ± 4.02	40.6 ± 5.39	< 0.001
BUN, mmol/L	6.41 ± 10.10	5.83 ± 6.74	7.67 ± 14.90	< 0.001
TG, mmol/L	2.05 ± 2.26	1.83 ± 1.59	2.50 ± 3.21	< 0.001
TC, mmol/L	4.82 ± 1.35	4.76 ± 1.22	4.95 ± 1.60	< 0.001
HDL-C, mmol/L	1.19 ± 0.36	1.21 ± 0.35	1.15 ± 0.36	< 0.001
LDL-C, mmol/L	2.66 ± 0.95	2.64 ± 0.90	2.71 ± 1.06	0.048
FT3, pmol/L	4.25 ± 1.01	4.31 ± 1.02	4.14 ± 0.97	< 0.001
FT4, pmol/L	13.9 ± 2.92	13.8 ± 2.47	13.9 ± 3.66	0.455
Urine Cr, g/L	1.14 [0.73, 1.75]	1.24 [0.80, 1.90]	0.96 [0.60, 1.47]	< 0.001
hs-CRP, mg/L	1.60 [0.70, 3.70]	1.40 [0.60, 3.10]	2.15 [0.90, 4.80]	< 0.001
mAlb, mg/L	19.7 [10.8, 58.4]	11.9 [10.8, 21.9]	118 [62.0, 279]	< 0.001
ACR, µg/mg	15.1 [12.5, 43.3]	12.5 [10.1, 15.7]	93.7 [47.0, 318]	< 0.001
Serum Cr, µmol/L	66.6 [56.1, 78.9]	65.4 [56.0, 75.9]	71.2 [57.0, 89.7]	< 0.001
eGFR, mL/min/1.73m^2^	115 [93, 138]	118 [99, 138]	106 [76, 136]	< 0.001
Comorbidity				
Hypertension	1614 (37.1)	933 (31.3)	681 (49.7)	< 0.001
Hyperlipidemia	979 (22.5)	656 (22.0)	323 (23.6)	0.257
Coronary heart disease	226 (5.2)	140 (4.7)	86 (6.3)	0.033
Stroke	178 (4.1)	98 (3.3)	80 (5.8)	< 0.001
Peripheral vascular disease	61 (1.4)	46 (1.6)	15 (1.1)	0.241
Cardiometabolic indices				
AIP	0.12 [-0.09, 0.35]	0.09 [-0.11, 0.31]	0.19 [-0.03, 0.45]	< 0.001
SHR	0.81 [0.67, 0.94]	0.81 [0.67, 0.93]	0.82 [0.65, 0.97]	0.030
TyG index	9.22 [8.72, 9.78]	9.12 [8.66, 9.64]	9.46 [8.91, 10.10]	< 0.001
HOMA-IR	3.06 [1.85, 5.17]	2.80 [1.70, 4.67]	3.87 [2.36, 6.44]	< 0.001
Medications				
CVD medication				
ACEI/ARB	406 (9.3)	213 (7.1)	193 (14.0)	< 0.001
Beta blocker	84 (1.9)	57 (1.9)	27 (1.9)	0.999
Statin	836 (19.2)	551 (18.5)	285 (20.7)	0.092
Glucose-lowering therapy				
Insulin	823 (18.9)	495 (16.6)	328 (23.8)	< 0.001
Metformin	1410 (32.4)	955 (32.0)	455 (33.0)	0.532
SGLT2i	651 (14.9)	390 (13.1)	261 (18.9)	< 0.001
GLP-1RA	170 (3.9)	97 (3.2)	73 (5.3)	0.002
DDP4i	549 (12.6)	381 (12.8)	168 (12.2)	0.625

The data are expressed as the mean ± standard deviation, median (interquartile range) or n (%)

ACEI, angiotensin-converting enzyme inhibitors; AIP, atherogenic index of plasma; ARB, angiotensin receptor blocker; BMI, body mass index; Cr, creatinine; DBP, diastolic blood pressure; eGFR, estimated glomerular filtration; FPG, fasting plasma glucose; FT3, free triiodothyronine; FT4, free thyroxine; HDL-C, high-density lipoprotein cholesterol; HGB, hemoglobin; hs-CRP, hypersensitive C-reactive protein; HOMA-IR, homeostasis model assessment of insulin resistance; LDL-C, low-density lipoprotein cholesterol; SBP, systolic blood pressure; SGLT2i, sodium–glucose cotransporter-2 inhibitors; SHR, stress hyperglycemia ratio; TC, total cholesterol; TG, triglycerides; GLP-1 RA, glucagon-like peptide 1 receptor agonists; DDP-4i, dipeptidyl-peptidase 4 inhibitor; TyG, triglyceride-glucose

### AIP and DKD

**Fig. 2:**
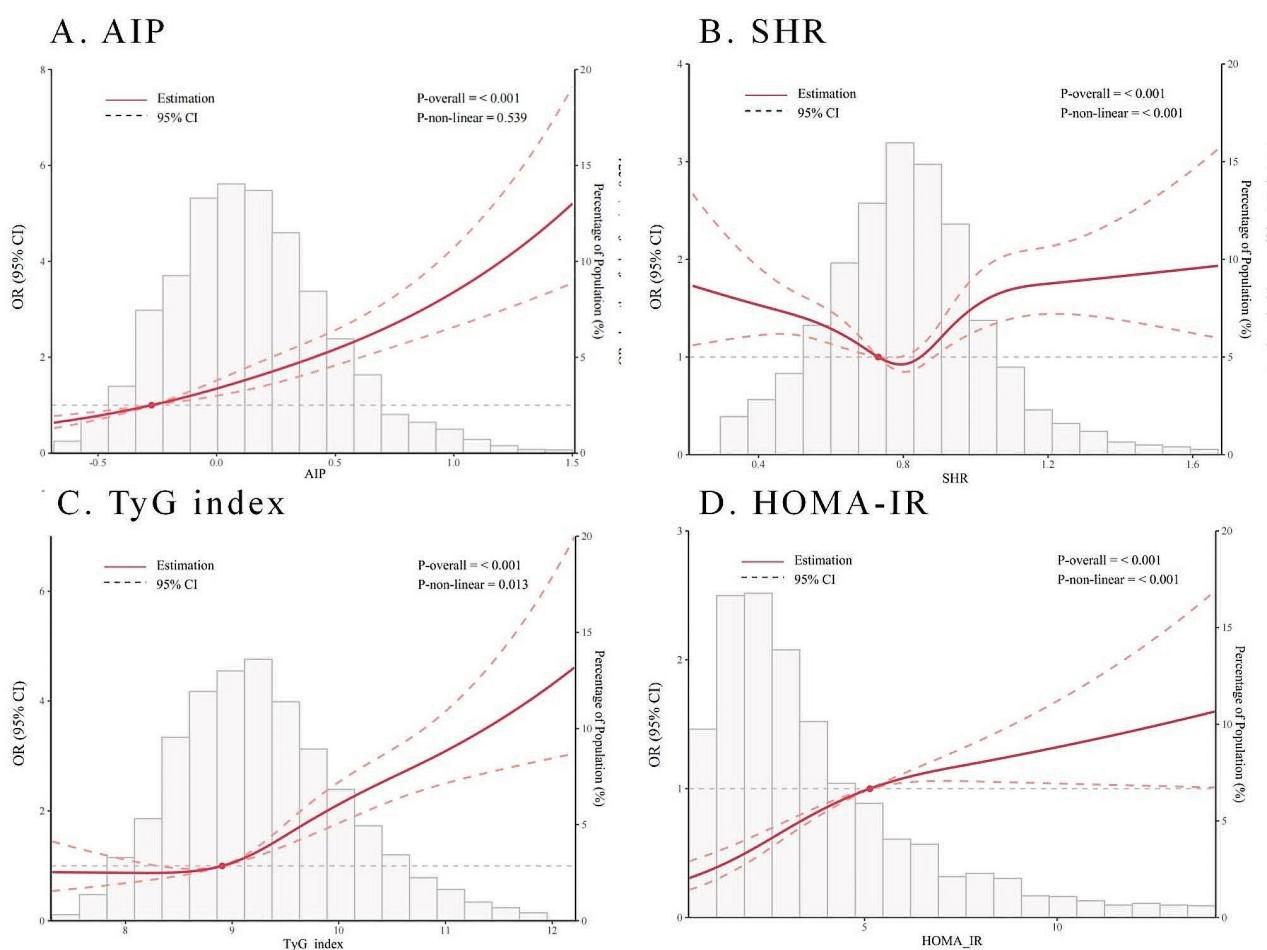
Nonlinear associations of the four cardiometabolic indices with different DKD in T2D patients (A) AIP; (B) SHR; (C) TyG index; (D) HOMA-IR; AIP, atherogenic index of plasma; SHR, stress–hyperglycemia ratio; TyG, triglyceride–glucose; HOMA–IR, homeostasis model assessment of insulin resistance

**Fig. 3:**
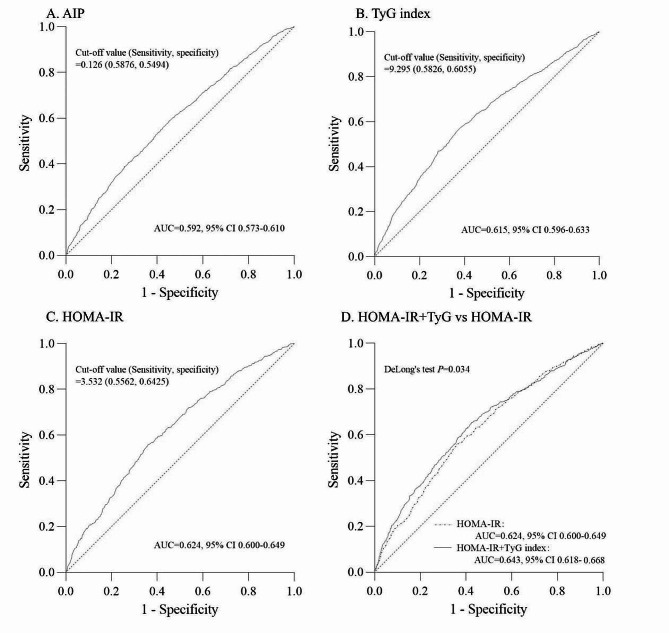
Receiver operating characteristic curves showing the performance of the AIP (A), the TyG index (B), and the HOMA-IR (C) and comparing the HOMA-IR combined with the TyG index to the HOMA-IR alone (D) for predicting DKD incidence AUC, area under the curve; AIP, atherogenic index of plasma; TyG, triglyceride-glucose; HOMA-IR, homeostasis model assessment of insulin resistance

Restricted cubic splines (RCSs) analysis (Fig. 2A) revealed a J-shaped association between the AIP and the risk of DKD. Table 2 displays the results of three multivariate logistic regression models evaluating the correlations between the AIP and DKD incidence. According to all three models, the highest AIP tertile was linked to an increased incidence of DKD (Model 1: OR 2.17, 95% CI 1.83–2.57, *P* < 0.001; Model 2: OR 1.59, 95% CI 1.22–2.08, *P* = 0.001; Model 3: OR 1.08, 95% CI 1.02–1.14, *P* = 0.005). The AIP, treated as a continuous variable, also exhibited a significant association with DKD incidence (Model 1: OR 1.09, 95% CI 1.06–1.12, *P* < 0.001; Model 2: OR 1.09, 95% CI 1.05–1.13, *P* < 0.001; Model 3: OR 1.08, 95% CI 1.04–1.12, *P* < 0.001). Subgroup analysis demonstrated consistent associations of the AIP with the risk of DKD across the age, sex, BMI, hyperlipidemia, and hypertension subgroups. However, this association became nonsignificant in patients without coronary heart disease (CHD) (Supplemental Table 1). ROC curve analysis revealed an optimal AIP cutoff value of 0.126 (sensitivity 58.76%, specificity 54.94%), with an AUC of 0.592 (95% CI = 0.573–0.610) (Fig. 3A).

### SHR and DKD

Figure 2B illustrates a clear U-shaped association between the SHR and the incidence of DKD. The SHR corresponding to the lowest risk of DKD according to multivariate-adjusted RCS analyses was 0.78. Compared to patients in the 1st tertile, those in the 3rd tertile exhibited a significantly greater risk of DKD (model 1: OR 1.30, 95% CI 1.11–1.51, *P* = 0.001; model 2: OR 1.30, 95% CI 1.03–1.64, *P* = 0.028; model 3: OR 1.42, 95% CI 1.12–1.81, *P* = 0.004; Table 2). When treated as a continuous variable, the SHR also demonstrated a significant association with DKD incidence (Model 1: OR 1.46, 95% CI 1.14–1.88, *P* = 0.003; Model 2: OR 1.60, 95% CI 1.13–2.27, *P* = 0.008; Model 3: OR 1.63, 95% CI 1.14–2.32, *P* = 0.007; Table 2). Subgroup analyses indicated that age, BMI, CHD incidence, hyperlipidemia, and hypertension influenced these associations. The associations remained significant for patient age < 65 years, BMI ≥ 28 kg/m², CHD, without hyperlipidemia, and without hypertension (Supplemental Table 2).

### TyG index and DKD

The RCS curve for the TyG index initially remained constant and then rapidly increased when the TyG index was > 8.9 (Fig. 2C). According to the multivariate logistic regression analysis, compared to patients in the 1st tertile of the TyG index, those in the 3rd tertile had a significantly greater incidence of DKD (model 1: OR 2.60, 95% CI 2.19–3.10, *P* < 0.001; model 2: OR 2.00, 95% CI 1.53–2.61, *P* < 0.001; model 3: OR 1.86, 95% CI 1.42–2.45, *P* < 0.001; Table 2). Similar results were observed when the TyG index was used as a continuous variable (Model 1: OR 1.79, 95% CI 1.64–1.95, *P* < 0.001; Model 2: OR 1.70, 95% CI 1.50–1.93, *P* < 0.001; Model 3: OR 1.62, 95% CI 1.43–1.84, *P* < 0.001). According to our subgroup analyses, the TyG index was associated with a high incidence of DKD, and this association was consistent across subgroups stratified by age, sex, BMI, CHD, hyperlipidemia, and hypertension (Supplemental Table 3). Moreover, there were no interactions between the TyG index and any of the other variables in the subgroup analyses (all P values for interaction > 0.05). According to the ROC curves for the entire study population, the optimal cutoff value for the TyG index was 9.295 (sensitivity 58.26%, specificity 60.55%). The AUC of the TyG index was 0.615 (95% CI = 0.596–0.633) (Fig. 3B).

**Table 2:** Logistic regression models for the association of cardiometabolic biomarkers with diabetic kidney disease

	AIP				SHR				TyG index				HOMA-IR		
	OR	95% CI	P value		OR	95% CI	P value		OR	95% CI	P value		OR	95% CI	P value
Model 1															
per unit increase	1.09	1.06–1.12	< 0.001		1.46	1.14–1.88	0.003		1.79	1.64–1.95	< 0.001		1.07	1.05–1.09	< 0.001
Tertile 1	Reference				Reference				Reference				Reference		
Tertile 2	1.31	1.10–1.56	0.003		0.84	0.71-1.00	0.048		1.20	1.00-1.45	0.054		1.60	1.25–2.05	< 0.001
Tertile 3	2.17	1.83–2.57	< 0.001		1.30	1.11–1.51	0.001		2.60	2.19–3.10	< 0.001		2.95	2.32–3.75	< 0.001
P for trend			< 0.001				0.005				0.004				< 0.001
Model 2															
per unit increase	1.09	1.05–1.13	< 0.001		1.60	1.13–2.27	0.008		1.70	1.50–1.93	< 0.001		1.04	1.02–1.07	0.001
Tertile 1	Reference				Reference				Reference				Reference		
Tertile 2	0.98	0.76–1.25	0.855		0.98	0.76–1.25	0.855		1.01	0.76–1.34	0.949		1.27	0.88–1.84	0.206
Tertile 3	1.59	1.22–2.08	0.001		1.30	1.03–1.64	0.028		2.00	1.53–2.61	< 0.001		2.47	1.70–3.59	< 0.001
P for trend			0.002				0.003				0.006				0.005
Model 3															
per unit increase	1.08	1.04–1.12	< 0.001		1.63	1.14–2.32	0.007		1.62	1.43–1.84	< 0.001		1.03	1.01–1.06	0.009
Tertile 1	Reference				Reference				Reference				Reference		
Tertile 2	1.00	0.95–1.06	0.968		1.15	0.89–1.48	0.295		1.01	0.76–1.35	0.944		1.25	0.86–1.81	0.252
Tertile 3	1.08	1.02–1.14	0.005		1.42	1.12–1.81	0.004		1.86	1.42–2.45	< 0.001		2.24	1.52–3.30	< 0.001
P for trend			0.014				0.012				0.009				0.023

AIP, atherogenic index of plasma; CI, confidence interval; HOMA-IR, homeostasis model assessment of insulin resistance; OR, odds ratio; SHR, stress hyperglycemia ratio; TyG, triglyceride-glucose

### HOMA-IR and DKD

The risk of DKD exhibited a significant increase with increasing HOMA-IR values, with the slope of this change becoming more gradual when HOMA-IR > 5 (Fig. 2D). According to the multivariate logistic regression analysis, compared to patients in the 1st tertile of HOMA-IR, those in the 3rd tertile had a more than 2-fold greater incidence of DKD (model 1: OR 2.95, 95% CI 2.32–3.75, *P* < 0.001; model 2: OR 2.47, 95% CI 1.70–3.59, *P* < 0.001; model 3: OR 2.24, 95% CI 1.52–3.30, *P* < 0.001). Subgroup analyses revealed a consistent association between HOMA-IR and a high incidence of DKD across various subgroups, including age, sex, BMI, coronary heart disease (CHD), hyperlipidemia, and hypertension (Supplemental Table 4). Furthermore, no interactions were detected between HOMA-IR and these variables in subgroup analyses (all P values for interaction > 0.05). According to the ROC curves for the entire study population, the optimal cutoff value for HOMA-IR was 3.532 (sensitivity 55.62%, specificity 64.25%). The area under the curve (AUC) of HOMA-IR was 0.624 (95% CI = 0.600-0.649; Fig. 3C). The HOMA-IR presented better predictive value than did the AIP and TyG index (Delong’s test: HOMA-IR vs. AIP, *P* = 0.012; HOMA-IR vs. TyG index, *P* = 0.039). Figure 3D demonstrates the enhanced predictive accuracy when HOMA-IR was added to the TyG index. The AUROC of the HOMA-IR combined with the TyG index for predicting DKD exhibited significantly greater sensitivity and specificity than did the AUC of the HOMA-IR alone (0.643; 95% CI = 0.618–0.668 vs. 0.624; 95% CI = 0.600–0.649; DeLong’s test *P* = 0.034).

### Cardiometabolic indices and hs-CRP

Table 3 illustrates the associations between the biomarkers (AIP, SHR, TyG index, and HOMA-IR) and hs-CRP. Spearman correlation analysis revealed significant positive correlations between hs-CRP and the four indices (AIP: coefficient = 0.295, *P* < 0.001; SHR: coefficient = 0.042, *P* = 0.049; TyG index: coefficient = 0.246, *P* < 0.001; HOMA-IR: coefficient = 0.339, *P* < 0.001). After controlling for age, BMI and SBP, partial correlation analysis revealed significant positive correlations between AIP, the TyG index and HOMA-IR with hs-CRP (AIP: coefficient = 0.181, *P* < 0.001; TyG index: coefficient = 0.183, *P* < 0.001; HOMA-IR: coefficient = 0.193, *P* < 0.001). The positive partial correlation between hs-CRP and the SHR remained weak and statistically nonsignificant (coefficient = 0.052, *P* = 0.083).

**Table 3:** The relationship between cardiometabolic biomarkers and high-sensitivity C-reactive protein (hs-CRP) as determined by Spearman’s test and partial correlation analysis

	AIP		SHR		TyG index		HOMA-IR	
Test method	Coefficient	P	Coefficient	P	Coefficient	P	Coefficient	P
Spearman correlation	0.295	< 0.001	0.042	0.049	0.246	< 0.001	0.339	< 0.001
Partial correlation	0.181	< 0.001	0.052	0.083	0.183	< 0.001	0.193	< 0.001

## Discussion

Our study was based on a prospective investigation including 4351 patients with T2D that was designed to evaluate the association between glucose and lipid metabolism disorders and the risk of DKD. The AIP was used to evaluate the atherogenicity of blood lipids, the TyG index and HOMA-IR were used to assess insulin resistance, and the SHR was used to evaluate the transient glucose fluctuations caused by psychological or physiological stress. Our study revealed that all four indices were independent predictors of DKD in T2D patients. Among the indices, the HOMA-IR had the strongest association with DKD and presented the best predictive accuracy. In addition, the combination of the HOMA-IR and TyG indices had a greater ability to predict DKD in T2D patients than did HOMA-IR alone. Therefore, it is important to comprehensively assess patients’ lipid and glucose metabolic status using tools such as the AIP, SHR, TyG index, and HOMA-IR to achieve better risk stratification and avoid diabetic complications.

The AIP, a simple and accessible indicator, combines HDL-C and TG concentrations for more comprehensive insight into dyslipidemia. Previous research has investigated the relationship between the AIP and the progression of IR and T2D. A cross-sectional study revealed that the AIP had an inverse L-shaped association with IR and a J-shaped association with T2D, indicating that the AIP should be reduced to a certain extent to prevent IR and T2D [8]. Another study revealed that a higher AIP was significantly associated with an increased incidence of prediabetes and diabetes in women [13]. A meta-analysis highlighted the closer association of the AIP with the risk of T2DM than traditional lipid parameters [14]. Recent studies suggest that the AIP may serve as a quantitative measure of small dense low-density lipoprotein (sdLDL) particles [15]. Characterized by challenges in clearance, susceptibility to oxidation, and easy uptake by macrophages leading to foam cell formation, sdLDL contributes to an increased risk of microvascular complications [16]. Despite the clinical limitations associated with the intricate and costly measurement of sdLDL [17], the novel lipid indicator AIP has emerged as a potentially more effective marker for assessing vascular risk. However, studies focusing on the association between the AIP and kidney damage in T2D patients have yielded controversial conclusions. For instance, Xu et al. [18] reported a positive association between the AIP and both the occurrence and severity of diabetic nephropathy (DN). Another study by Qi et al. [19] involving 335 Chinese patients identified the AIP as an independent risk factor for microalbuminuria in newly diagnosed T2DM patients. Conversely, a study with 2523 T2D patients found no significant difference in DN incidence among AIP tertiles [20]. Therefore, our study utilized a large cohort to provide additional evidence that an increased AIP is associated with a greater risk of DKD independent of other risk factors and medication.

The TyG index and HOMA-IR have been proposed as surrogate markers for metabolic syndrome and insulin resistance [10]. Substantial evidence has validated the crucial role of the TyG index and HOMA-IR in predicting macrovascular disease [21–23]. However, studies on the correlation between the TyG index or HOMA-IR and DKD incidence are insufficient. A study involving 682 Chinese patients with type 2 diabetes revealed that an elevated TyG index was an independent risk factor (OR 1.91, *P* = 0.001) for diabetic nephropathy, defined as an albumin excretion rate > = 30 mg/day or > = 20 µg/min [24]. Another study, which included 1413 patients, reported that the presence of nephropathy was linked to a higher TyG index (OR = 1.703, *P* < 0.001). Data from the RADAR and SONAR trials, which enrolled participants with type 2 diabetes and chronic kidney disease (CKD), demonstrated that an increase in HOMA-IR was associated with an elevated risk of composite cardiorenal outcomes and kidney-related outcomes [25]. In the present study, we identified a notable positive correlation between the TyG index and HOMA-IR with DKD. Compared with the findings of previous studies, the present study was strengthened by a large sample size, and we calculated that the optimal cutoff values of the TyG index and HOMA-IR for predicting DKD were 9.295 and 3.532, respectively. In addition, we found that the predictive value of the AUCROC of HOMA-IR was greater than that of the TyG index. Additionally, the AUCROC of the HOMA-IR combined with the TyG index for predicting DKD exhibited significantly greater sensitivity and specificity than did the AUC of the HOMA-IR alone for type 2 diabetes patients. The mechanism driving this relationship was attributed to the influence of insulin resistance on renal structure. One hypothesis is that insulin resistance may be associated with elevated glomerular hydrostatic pressure, leading to increased renal vascular permeability and, ultimately, glomerular hyperfiltration [26]. In addition, metabolic changes associated with insulin resistance lead to glomerular hypertrophy, glomerulosclerosis, tubulointerstitial inflammation and fibrosis [27, 28].

Stress hyperglycemia refers to an acute increase in blood glucose levels caused by physiological or psychological stress [29]. The SHR, which assesses the extent of stress-related hyperglycemia in relation to the severity of illness, has been proposed as a potential indicator for predicting unfavorable outcomes in critically ill individuals, such as those with acute myocardial infarction [29, 30], heart failure [5, 9, 31, 32], and ischemic stroke [33, 34]. Recent research has also revealed that the SHR serves as an indicator of the severity of acute kidney injury in patients with CVD. For instance, a recently published study reported a significant association between both the lowest and highest fasting SHR and an increased occurrence of contrast-induced acute kidney injury in individuals undergoing coronary angiography or percutaneous coronary intervention [35]. Additionally, a U-shaped relationship was detected between SHR and acute kidney injury in patients with heart failure [9, 31]. In the present study, through RCS analysis, we observed a U-shaped relationship between SHRs and DKD in patients with type 2 diabetes. Both high and low SHR were associated with an increased risk of DKD, consistent with findings from previous studies. The association between SHR and DKD can be largely attributed to the inflammatory response triggered by blood glucose fluctuations [36]. A rapid increase in blood glucose leads to an overproduction of reactive oxygen species (ROS) in the endothelial cells of renal blood vessels. Oxidative stress can result in endothelial dysfunction and impaired vasodilation [37]. Furthermore, stress hyperglycemia often leads to impaired fibrinolysis through increased levels of plasminogen activator inhibitor-1 (PAI-1), which contributes to the formation of additional blood clots in vessels [38]. Conversely, a low SHR indicates the occurrence of hypoglycemic episodes due to unsuitable intensive blood glucose control, which is also harmful to the subject [39].

**Fig. 4:**
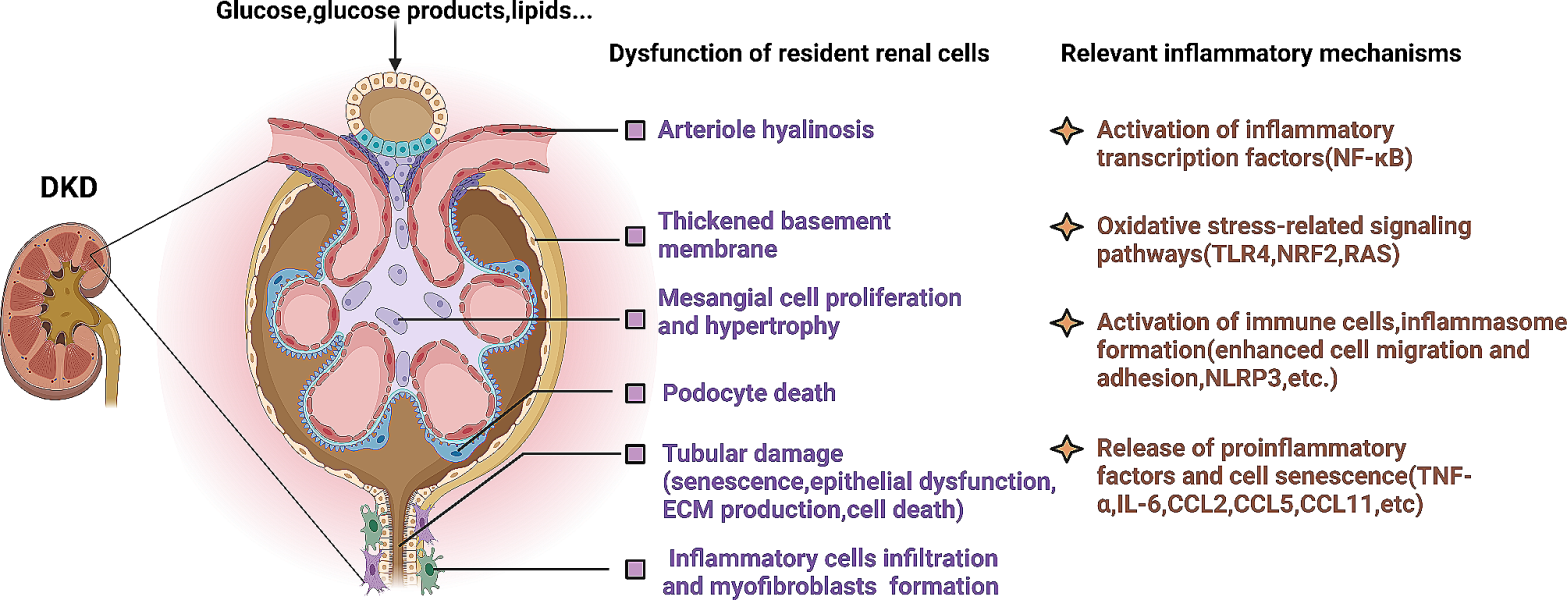
This figure (created with BioRender.com) provides a comprehensive overview of the relationship between DKD and inflammation. Under the stimulation of glucose and fatty acids, various cells within the glomeruli and renal tubules (such as endothelial cells, mesangial cells, podocytes, inflammatory cells, fibroblasts, etc.) undergo pathological changes associated with inflammation. These changes involve the activation of inflammation-related transcription factors and downstreampathways, as well as the release of inflammatory factors and cellular senescence. The abbreviations used in the figure are as follows: NF-κB, nuclear factor kappa-B; TLR4, Toll-like receptor 4; NRF2, nuclear factor erythroid 2-related factor 2; RAS, renin-angiotensin system; NLPR3, NOD-like receptor family pyrin domain-containing 3; TNF-α, tumor necrosis factor-α; IL-6, interleukin 6; and CCL2/CCL5/CCL11, chemokine (CC-motif) ligand 2/5/11.

Inflammation serves as a pivotal factor in the interplay between metabolic syndrome and kidney damage. [40, 41] High levels of glucose and glucose-derived products, as well as lipids, can contribute to glomerular damage, such as mesangial proliferation, collagen deposition, podocyte loss, and hypertrophy. Additionally, these factors can also lead to tubular damage, including cellular senescence, epithelial atrophy, and myofibroblast activation. Immune cells infiltrate the kidney through these processes [42, 43]. We delineated the possible pathological changes that occur in different resident renal cells of DKD samples in the presence of hyperglycemia- or lipid-induced inflammation compared to healthy controls (Fig. 4). These findings may provide insights into potential strategies for targeted interventions for DKD in the future.

In our study, we discovered a strong correlation between the AIP, TyG index, and HOMA-IR and the inflammation biomarker hs-CRP. In addition to our study, several other studies have discussed the roles of inflammation and oxidative stress in DKD development. For example, Hassannejad et al. [40] reported a significant association between IL-6 and CRP levels and the risk of metabolic syndrome. Fatty Zucker (ZF) rats fed for 2 months and obese mice fed a high-fat diet for 5 months have been reported to have significantly greater body weight and albuminuria than their controls [44]. In these mice, inflammatory markers such as TNF-α, the chemokine C-C-motif receptor 2 (CCR2), and nuclear factor kappa-B (NF-κB) are elevated or activated in the glomeruli, indicating a close correlation between CKD, inflammation, and oxidative stress [44]. Furthermore, a diabetic state and insulin resistance can lead to the overproduction of reactive oxygen species (ROS), activating diacylglycerol (DAG)-protein kinase C (PKC) signaling and causing the accumulation of extracellular matrix in the glomeruli, ultimately contributing to the progression of DKD [45–47]. Several therapeutic drugs targeting oxidative stress and inflammation are used for DKD treatment, including metformin, sodium-glucose cotransporter 2 (SGLT2) inhibitors, imeglimin, aspirin, cyclooxygenase-2 inhibitors, olmesartan, mineralocorticoid receptor antagonists (MRAs), and incretin-based agents. These drugs mainly focus on restoring mitochondrial function, reducing ROS levels, and other mechanisms [42, 47–49]. Additionally, endogenous protective factors such as antioxidant enzymes, insulin, and vascular endothelial growth factor are suggested to be involved in the prevention of diabetic nephropathy [48].

Our study showed that both lipid and glucose metabolic disorders were associated with microvascular complications in T2D patients and are highly important for clinical treatment. In addition, we calculated the optimal cutoff values for the indices for DKD incidence. Patients with an AIP > 0.126, a TyG index > 9.295 or a HOMA-IR > 3.532 were at greater risk of DKD than other patients were. Clinically, the use of these indices for patient assessment could help achieve more comprehensive and accurate risk stratification in T2D patients, which can help clinicians provide appropriate treatment and nursing care levels to reduce rates of renal complications and reduce medication costs. However, it is imperative to acknowledge the limitations inherent in our study. First, the research was limited to a single center, underscoring the necessity for additional validation across multiple centers to bolster the robustness and generalizability of our findings. Second, our study could not monitor long-term changes in the four cardiometabolic indices or the progression of DKD. Given the observational nature of our study, establishing a direct causal relationship between cardiometabolic indices and DKD based solely on the results obtained is unfeasible. Third, despite our best efforts to include a wide range of covariates and potential confounding factors in our analysis, importantly, there might still be unmeasured variables such as genetic factors, dietary patterns, psychosocial factors and health care disparities.

## Conclusions

This study explored the associations between four cardiometabolic indices and DKD in T2D patients. Our findings revealed that increased AIP, TyG index and HOMA-IR were associated with a greater risk of DKD, while both low and high SHR were associated with an elevated risk of DKD. In addition, the optimal cutoff values for the AIP, TyG index and HOMA-IR were 0.126, 9.259 and 3.532, respectively. In addition, among these indices, the HOMA-IR score exhibited the strongest association with and predictive value for DKD.

### Electronic supplementary material

Below is the link to the electronic supplementary material.

Supplementary Material 1

## Data Availability

No datasets were generated or analysed during the current study.

## Abbreviations

ACEI: angiotensin-converting enzyme inhibitors
AIP: atherogenic index of plasma
ARB: angiotensin receptor blocker
BMI: body mass index
Cr: creatinine
DBP: diastolic blood pressure
FPG: fasting plasma glucose
FT3: free triiodothyronine
FT4: free thyroxine
HDL-C: high-density lipoprotein cholesterol
HGB: hemoglobin
HOMA-IR: homeostasis model assessment of insulin resistance
LDL-C: low-density lipoprotein cholesterol
SGLT2i: sodium–glucose cotransporter-2 inhibitors
SHR: stress hyperglycemia ratio
TC: total cholesterol
TG: triglycerides
TyG: triglyceride–glucose
GLP-1 RA: glucagon-like peptide 1 receptor agonists
DDP4i: dipeptidyl-peptidase 4 inhibitor
