# Supplementary material for: Associations between cardiometabolic indices and the risk of diabetic kidney disease in patients with type 2 diabetes
Source: Cardiovasc Diabetol. 2024 Apr 25;23:142. doi: 10.1186/s12933-024-02228-9 (PMC11046854; doi:10.1186/s12933-024-02228-9)
Supplement: Supplementary file 1 — Supplementary Material 1 [file 12933_2024_2228_MOESM1_ESM.docx]

Supplementary Table 1. Subgroup analyses of the association between atherogenic index of plasma (AIP) and diabetic kidney disease according to patients characteristics and comorbidities.

| Subgroup | **beta** | **se** | **OR** | 95% CI | P-value | Pinteraction |
| --- | --- | --- | --- | --- | --- | --- |
| Age, year |  |  |  |  |  |  |
| <65 | 0.0733 | 0.0127 | 1.08 | 1.05-1.10 | <0.001 | 0.263 |
| ≥65 | 0.1068 | 0.0518 | 1.11 | 1.01-1.23 | 0.039 |  |
| Gender |  |  |  |  |  |  |
| Male | 0.1068 | 0.0518 | 1.11 | 1.01-1.23 | 0.039 | 0.065 |
| Female | 0.1103 | 0.0277 | 1.12 | 1.06-1.18 | <0.001 |  |
| BMI |  |  |  |  |  |  |
| <28 kg/m2 | 0.0573 | 0.014 | 1.06 | 1.03-1.09 | <0.001 | 0.437 |
| ≥28 kg/m2 | 0.0646 | 0.0255 | 1.07 | 1.01-1.12 | 0.011 |  |
| **Coronary heart disease** |  |  |  |  |  |  |
| Yes | 0.1213 | 0.0262 | 1.13 | 1.07-1.19 | <0.001 | 0.003 |
| No | 0.0377 | 0.0205 | 1.04 | 1.00-1.08 | 0.066 |  |
| **Hyperlipidemia** |  |  |  |  |  |  |
| Yes | 0.0899 | 0.0289 | 1.09 | 1.03-1.16 | 0.002 | 0.632 |
| No | 0.0752 | 0.0236 | 1.08 | 1.03-1.13 | 0.002 |  |
| **Hypertension** |  |  |  |  |  |  |
| Yes | 0.0872 | 0.0299 | 1.09 | 1.03-1.16 | 0.004 | 0.657 |
| No | 0.0724 | 0.0217 | 1.08 | 1.03-1.12 | 0.001 |  |

*CI* confidence interval, *OR* odds ratio.

Supplementary Table 2. Subgroup analyses of the association between stress hyperglycemia ratio (SHR) and diabetic kidney disease according to patients characteristics and comorbidities.

| Subgroup | **beta** | **se** | **OR** | 95% CI | P-value | Pinteraction |
| --- | --- | --- | --- | --- | --- | --- |
| Age, year |  |  |  |  |  |  |
| <65 | 0.4346 | 0.148 | 1.54 | 1.16-2.06 | 0.003 | 0.043 |
| ≥65 | 0.5202 | 0.3863 | 1.68 | 0.79-3.59 | 0.178 |  |
| Gender |  |  |  |  |  |  |
| Male | 0.5021 | 0.2019 | 1.65 | 1.11-2.45 | 0.013 | 0.146 |
| Female | 0.7490 | 0.3584 | 2.11 | 1.05-4.27 | 0.037 |  |
| BMI |  |  |  |  |  |  |
| <28 kg/m2 | 0.1497 | 0.1434 | 1.16 | 0.88-1.54 | 0.296 | 0.032 |
| ≥28 kg/m2 | 1.4075 | 0.4635 | 4.09 | 1.65-10.13 | 0.002 |  |
| **Coronary heart disease** |  |  |  |  |  |  |
| Yes | 0.5106 | 0.2304 | 1.67 | 1.06-2.62 | 0.027 | 0.008 |
| No | 0.4897 | 0.2821 | 1.63 | 0.94-2.84 | 0.083 |  |
| **Hyperlipidemia** |  |  |  |  |  |  |
| Yes | 0.5449 | 0.5011 | 1.72 | 0.65-4.60 | 0.277 | 0.029 |
| No | 0.4943 | 0.1905 | 1.64 | 1.13-2.38 | 0.010 |  |
| **Hypertension** |  |  |  |  |  |  |
| Yes | 0.3665 | 0.2801 | 1.44 | 0.83-2.50 | 0.191 | 0.043 |
| No | 0.6199 | 0.2311 | 1.86 | 1.18-2.92 | 0.007 |  |

*CI* confidence interval, *OR* odds ratio.

Supplementary Table 3. Subgroup analyses of the association between triglyceride-glucose (TyG) index and diabetic kidney disease according to patients characteristics and comorbidities.

| Subgroup | **beta** | **se** | **OR** | 95%CI | P-value | Pinteraction |
| --- | --- | --- | --- | --- | --- | --- |
| Age, year |  |  |  |  |  |  |
| <65 | 0.5575 | 0.0469 | 1.75 | 1.59-1.91 | <0.001 | 0.753 |
| ≥65 | 0.4691 | 0.0974 | 1.60 | 1.32-1.93 | <0.001 |  |
| Gender |  |  |  |  |  |  |
| Male | 0.4691 | 0.0974 | 1.60 | 1.32-1.93 | <0.001 | 0.589 |
| Female | 0.5778 | 0.0752 | 1.78 | 1.54-2.07 | <0.001 |  |
| BMI |  |  |  |  |  |  |
| <28 kg/m2 | 0.4799 | 0.0479 | 1.62 | 1.47-1.77 | <0.001 | 0.423 |
| ≥28 kg/m2 | 0.7497 | 0.1548 | 2.12 | 1.56-2.87 | <0.001 |  |
| **Coronary heart disease** |  |  |  |  |  |  |
| Yes | 0.5363 | 0.0823 | 1.71 | 1.45-2.01 | <0.001 | 0.089 |
| No | 0.3829 | 0.1110 | 1.47 | 1.18-1.82 | 0.001 |  |
| **Hyperlipidemia** |  |  |  |  |  |  |
| Yes | 0.6137 | 0.1403 | 1.85 | 1.40-2.43 | <0.001 | 0.647 |
| No | 0.4585 | 0.0751 | 1.58 | 1.37-1.83 | <0.001 |  |
| **Hypertension** |  |  |  |  |  |  |
| Yes | 0.3522 | 0.1061 | 1.42 | 1.16-1.75 | 0.001 | 0.368 |
| No | 0.5494 | 0.0833 | 1.73 | 1.47-2.04 | <0.001 |  |

*CI* confidence interval, *OR* odds ratio.

Supplementary Table 4. Subgroup analyses of the association between homeostasis model assessment of insulin resistance (HOMA-IR) and diabetic kidney disease according to patients characteristics and comorbidities.

| Subgroup | **beta** | **se** | **OR** | 95%CI | P-value | Pinteraction |
| --- | --- | --- | --- | --- | --- | --- |
| Age, year |  |  |  |  |  |  |
| <65 | 0.0604 | 0.0104 | 1.06 | 1.04-1.08 | <0.001 | 0.834 |
| ≥65 | 0.0621 | 0.0187 | 1.06 | 1.03-1.10 | 0.001 |  |
| Gender |  |  |  |  |  |  |
| Male | 0.0621 | 0.0187 | 1.06 | 1.03-1.10 | 0.001 | 0.567 |
| Female | 0.0475 | 0.0122 | 1.05 | 1.02-1.07 | <0.001 |  |
| BMI |  |  |  |  |  |  |
| <28 kg/m2 | 0.0542 | 0.0109 | 1.06 | 1.03-1.08 | <0.001 | 0.298 |
| ≥28 kg/m2 | 0.1231 | 0.0339 | 1.13 | 1.06-1.21 | <0.001 |  |
| **Coronary heart disease** |  |  |  |  |  |  |
| Yes | 0.0456 | 0.0170 | 1.05 | 1.01-1.08 | 0.007 | 0.468 |
| No | 0.0527 | 0.0200 | 1.05 | 1.01-1.10 | 0.008 |  |
| **Hyperlipidemia** |  |  |  |  |  |  |
| Yes | 0.0472 | 0.0214 | 1.05 | 1.01-1.09 | 0.028 | 0.390 |
| No | 0.0596 | 0.0146 | 1.06 | 1.03-1.09 | <0.001 |  |
| **Hypertension** |  |  |  |  |  |  |
| Yes | 0.0572 | 0.0202 | 1.06 | 1.02-1.10 | 0.005 | 0.481 |
| No | 0.0559 | 0.0156 | 1.06 | 1.03-1.09 | <0.001 |  |

*CI* confidence interval, *OR* odds ratio.
